# Systemic Toxicity of L‐Mimosine in Rabbits: A Non‐Rodent Model for Safety Assessment

**DOI:** 10.1002/jat.4913

**Published:** 2025-09-04

**Authors:** S. M. Ferreira, H. Zapparoli, C. S. Mussi, P. C. Maiorka, A. F. C. Andrade, S. L. Górniak, A. T. Gotardo

**Affiliations:** ^1^ Research Centre for Veterinary Toxicology (CEPTOX) – Department of Pathology, School of Veterinary Medicine and Animal Sciences University of São Paulo Pirassununga SP Brazil; ^2^ Research Swine Center (NPS) – Department of Animal Reproduction, School of Veterinary Medicine and Animal Sciences University of São Paulo Pirassununga SP Brazil

**Keywords:** histopathology, lagomorph, *Leucaena leucocephala*, *Mimosa pudica*, phytotoxin

## Abstract

L‐mimosine is a non‐protein amino acid primarily found in the Mimosoideae subfamily, with high concentrations in *Leucaena leucocephala* and *Mimosa pudica* . These plants are widely used in both human and animal nutrition, as well as in phytotherapeutic applications. While the toxic effects of L‐mimosine have been extensively studied in ruminants, its impact on monogastric species remains unexplored. Given the widespread use of these plants and the limited knowledge regarding L‐mimosine toxicity in monogastric animals, this study aimed to investigate its toxicological effects in non‐rodent monogastric species by assessing its subacute toxicity. To achieve this, L‐mimosine was extracted from *L. leucocephala* seeds and administered orally, incorporated into the feed, at doses of 25, 40, and 60 mg/kg for 28 days in male rabbits. Although no clinical, biochemical, hormonal, or macroscopic alterations were observed, histopathological analyses revealed dose‐dependent lesions in the liver, kidneys, thyroid, and spleen. These findings suggest that rabbits may be particularly susceptible to the toxic effects of L‐mimosine.

## Abstract 2

This study investigated the subacute toxicity of L‐mimosine in male rabbits, a non‐rodent monogastric model. L‐mimosine, isolated from *Leucaena leucocephala* seeds, was incorporated into the diet and administered orally at doses of 25, 40, and 60 mg/kg for 28 days. While no clinical, biochemical, hormonal, or gross pathological changes were observed, histopathological examination revealed dose‐dependent lesions in the liver, kidneys, thyroid, and spleen, indicating a marked susceptibility of rabbits to L‐mimosine toxicity.

## 1 Introduction

L‐mimosine, a non‐protein amino acid primarily found in the Mimosoideae subfamily, is abundant in *Leucaena leucocephala* and *Mimosa pudica* , *L. leucocephala* is an invasive and highly adaptable species (Smith 1985) widespread across tropical and subtropical regions of Asia, Africa, and the Americas (Sharma et al. 2022). Its high protein content, good digestibility, and drought resilience (Andrew et al. 2000; Radostits et al. 2000) make it a valuable protein supplement for livestock (Garcia et al. 1996; D'Mello and Acamovic 1989; De Angelis et al. 2021) and a non‐conventional protein source for humans, consumed both fresh and processed (Bourges and De León 1980; Aquino‐González et al. 2023). Beyond its nutritional value, *Leucaena* exhibits diverse therapeutical pharmacological properties, including antimicrobial (Aderibigbe et al. 2011), anticancer (Dipe et al. 2012; Xu and Cai 2018), anthelmintic (Widaad et al. 2022), repellent (Meena 2013), and hypoglycemic effects (Sumarny and Simanjuntak 2010).

Likewise, *M. pudica* has been extensively studied for its medicinal, agricultural, and environmental applications, demonstrating antioxidant, antimicrobial, anti‐inflammatory, antidiabetic, anticancer, neuroprotective, anthelmintic, wound healing, and hepatoprotective activities, positioning it as a potential therapeutic agent for infectious, metabolic, and neurological disorders (Gunawardhana et al. 2015). In agriculture and veterinary medicine, it serves as a natural pesticide, nitrogen‐fixing soil enhancer, and potential fodder source, though excessive consumption may be toxic (Dos Reis et al. 2010; Mondol and Islam 2020). Environmentally, it contributes to bioremediation by absorbing heavy metals and pollutants, while its extensive root system aids in soil erosion prevention (Balamoorthy et al. 2022; Abdullahi et al. 2020).

The bioactive and therapeutic effects of *L. leucocephala* and *M. pudica* are largely attributed to L‐mimosine, highlighting its significance as a potential therapeutic compound in pharmacology, as well as its importance in veterinary medicine and environmental science.

Although L‐mimosine has applications in food and potential beneficial properties, its toxicity is well documented. Adverse effects in farm animals have been reported due to the ingestion of *L. leucocephala* or *M. pudica* . These effects include body weight loss, alopecia, goiter, abortion, cataracts, infertility, excessive salivation, mouth ulcers, poor growth, uterine perforation, and fetal deformation (Ram et al. 1994; Kamada et al. 1997; Yanuartono et al. 2019; Driehuis et al. 2018; Machado et al. 2024). Numerous in vitro and in vivo experimental studies have reproduced the harmful effects of L‐mimosine previously observed in livestock (Nguyen and Tawata 2016). Our research group demonstrated in rats the goitrogenic, reproductive, and immunotoxic effects of L‐mimosine (Gotardo et al. 2011; de Almeida et al. 2025; Hueza et al. 2023). Additionally, a review by Nguyen and Tawata (2016) suggests that L‐mimosine may contribute to neurodegenerative diseases, including Parkinson's disease.

Given the extensive use of *L. leucocephala* in human food and medicine, and considering that relative to ruminants, little is known about the impact of L‐mimosine in monogastric species, this study aims to further elucidate the toxicological effects of L‐mimosine in monogastric non‐rodent species by assessing its subacute toxicity. Compared to rodents, rabbits offer distinct advantages in sample collection, allowing for more comprehensive pharmacokinetic and toxicokinetic analyses and enabling precise tracking of toxicity progression. Their metabolism, particularly thyroid hormone metabolism, is more similar to that of humans, and their larger, more human‐like skin structure makes them a superior model for dermal toxicity studies (Sokolowski et al. 2024). In reproductive toxicology, rabbits provide greater physiological relevance, better control over reproductive variables, and enhanced experimental capabilities. Their placental similarity to humans, induced ovulation, feasibility for embryo culture, and the ability for repeated sampling make them an optimal model for assessing reproductive and developmental toxicants in preclinical research (Foote and Carney 2000).

Considering the limited data on L‐mimosine toxicity in humans, we used rabbits as an experimental model, administering this amino acid over a 28‐day period to improve understanding of its safety profile and generate more predictive data for human health risk assessment.

## 2 Material and Methods

This study was conducted at the University of Sao Paulo (USP). The procedures were approved by the USP Animal Ethics Committee (protocol number CEUA 8425111022), and all animal care and handling were performed by experienced personnel under veterinary supervision. All efforts were made to minimize animal suffering.

To avoid bias, all the experiments were performed in a blinded manner with respect to the treatments. After the randomized allocation of animals to the treatments (Zolman 1993), animals, samples, and treatments were coded until the data analysis.

### 2.1 Plant Material

*L. leucocephala* seeds were collected from plants cultivated at the Research Centre for Veterinary Toxicology (CEPTOX), University of São Paulo (USP), Pirassununga, Brazil (21°58′ S, 47°27′ W).

### 2.2 Extraction and Quantification of L‐Mimosine

The mimosine extraction procedures were carried out based on the methodology proposed by Hegarty et al. (1964), with adaptations. For the extraction, the cationic ion‐exchange resin Amberlite IR 100B was used. Prior to the extraction process, the resin was prepared as follows: after being packed into a glass column (1 m in length × 5 cm in diameter), it was equilibrated with a 4 N HCl solution overnight, using 100 mL of 4 N HCl for every 110 mL of resin. Subsequently, the cationic resin was converted to the Na^+^ form using a 2 N NaOH solution, followed by a 2 N HCl solution, both applied in a ratio of 100 mL for every 110 mL of resin. After these steps, the resin was thoroughly washed with distilled water (approximately 1.2 L for every 110 g of resin) until the pH of the water was between 6.0 and 7.0, rendering the resin ready for use.

A total of 880 mL of the pre‐treated resin and 800 g of *L. leucocephala* seeds were used. The resin was divided into eight equal portions (110 mL each) and placed into vegetable cellophane dialysis bags (25 × 25 cm), to which 6 g of corn starch were added to each bag. Simultaneously, a solution was prepared by combining 9.6 L of distilled water with 800 g of *L. leucocephala* seeds that had been previously ground into a fine powder using an industrial mill. After preparing the solution, four cellophane bags containing the resin were immersed in the mixture and kept under constant agitation on a magnetic stirrer for 24 h. Subsequently, the initial four bags were removed from the solution and replaced by the remaining four bags, which were left in the mixture under agitation for an additional 36 h.

After this period, the resin from all the bags was collected, transferred back into the column, and washed with 8 L of distilled water. The resin was then rinsed with 1.6 L of 80° ethanol solution to remove excess corn starch, followed by an additional wash with 16 L of distilled water. Mimosine was eluted from the resin using a 2 N ammonium hydroxide solution, applied in sufficient quantity to achieve complete elution, as indicated by a change in the eluate's color from yellowish to clear. The eluate was then collected, stored in an amber bottle, and concentrated using a rotary evaporator at 40°C.

After rotary evaporation, the precipitate was dissolved in 50 mL of distilled water, and the pH was adjusted to 4.5–5.0 using a 4 N HCl solution. At this stage, most of the extracted mimosine precipitated, resulting in the formation of a biphasic suspension. The suspension was homogenized and distributed into 50 mL Falcon tubes. The tubes were centrifuged at 4000 rpm for 10 min, and the supernatant was removed. Subsequently, an equal volume of distilled water was added, and the centrifugation process was repeated under the same conditions. The final supernatant was discarded. The resulting samples were frozen and later lyophilized to obtain mimosine in powdered form.

The samples were analyzed by thin‐layer chromatography (TLC) using Merck F254 silica gel plates mounted on a glass support. The mobile phase consisted of a solution of butanol, acetic acid, and water (BAW) in a 12:3:5 ratio, respectively. Plate development was performed using a 10% ferric chloride solution. For reference, a commercial mimosine standard from Sigma‐Aldrich with a purity greater than 98% was used.

To assess the purity of the extracted mimosine, high‐performance liquid chromatography (HPLC) with ultraviolet (UV) detection at 280 nm was performed, using a Sigma‐Aldrich mimosine standard with a purity greater than 98%. The HPLC procedure followed these steps: first, the mimosine standard was diluted to 1 mg/mL in 1 N HCl. From this solution, aliquots of 50, 40, 30, 20, and 10 μL were diluted in 1 mL of a 0.2% H_3_PO_4_ solution. The extracted mimosine samples were prepared identically to the standard. Subsequently, 5 μL volumes of each sample and the standard were analyzed by HPLC‐UV using a Hypercarb Thermo Scientific column. Peak areas were measured and recorded using the HPLC system software, and the degree of purity was calculated as a percentage.

### 2.3 Animals and Housing

Twenty healthy male New Zealand rabbits ( *Oryctolagus cuniculus* ), aged between 6 and 7 months and weighing between 2.6 ± 0.3 kg, were used. They were kept in the Research Center of Veterinary Toxicology (CEPTOX), Department of Pathology, School of Veterinary Medicine and Animal Science, University of São Paulo (FMVZ/USP). The animals were individually housed in suspended wire cages (80 × 60 × 40 cm) under controlled environmental conditions, including a room temperature of 22°C ± 2°C, humidity levels ranging from 45% to 65%, and a 12‐h light/dark cycle.

### 2.4 Preparation of L‐Mimosine‐Supplemented Feed and Feeding

The animals were provided with commercial species‐specific feed (Coelhão, Guabi). Water was supplied ad libitum through automatic drinkers.

L‐mimosine was administered orally to the animals, incorporated into the commercial feed. The incorporation process involved pre‐grinding the commercial feed and, after adding the L‐mimosine, homogenizing the mixture using a mixer (Marconi), following the recommendations of the American Society of Animal Science (ASAS 2011). Water (15% of the total weight) was then added to facilitate pelletization, which was performed using a pellet press (Chavantes). Finally, the pelleted feed, supplemented or not with mimosine, was provided to the animals.

Four feed formulations with different L‐mimosine concentrations were prepared: 0.0%, 0.10%, 0.16%, and 0.24% (equivalent to 0.0, 1.0, 1.6, and 2.4 g of L‐mimosine per kg of feed, respectively). The L‐mimosine inclusion percentages were calculated to ensure that each 25 g portion of feed contained 0.0, 25.0, 40.0, or 60.0 mg of L‐mimosine, depending on the established concentration. The amount of L‐mimosine incorporated into the feed was calculated based on the active content to ensure accurate dosing in accordance with the experimental design. During the experimental design, animals in each group were fed 25 g of L‐mimosine‐supplemented feed, specific to their group, per kilogram of body weight per day. After the complete consumption of this portion, the diet was supplemented with L‐mimosine‐free commercial feed in an amount sufficient to achieve a total daily intake of 120 g/kg of body weight.

### 2.5 Experimental Design

Male rabbits were randomly allocated into four treatment groups (*n* = 5 per group) and given daily the following doses of L‐mimosine orally in the feed, in mg per kg body weight (mg/kg BW): 0 mg (control group), 25 (MI25 group), 40 (MI40 group), and 60 (MI60 group). This regimen was followed for 28 consecutive days. Clinical observations were performed every other day to evaluate signs of intoxication and mortality. On Days 0, 7, 14, 21, and 28 of the experiment, the rabbits were weighed, and blood samples were collected from the radial ear vein using vacuum tubes, with anticoagulant, for a complete blood count analysis using Rayto HMG 51 Vet automatic equipment.

On Days 0, 14, and 28, an aliquot of blood was collected without anticoagulant for biochemical analysis. The serum was separated by allowing the blood (taken in a plain tube) to clot and then centrifuged at 4000 rpm for 10 min at 4°C. Serum biochemistry was analyzed using a Celm‐SBA200 biochemistry analyzer with specific commercial kits. The biochemical markers measured included glucose, albumin, total proteins, cholesterol, urea, and creatinine, which were quantified, along with the activity of the enzymes gamma‐glutamyl transferase (GGT), aspartate aminotransferase (AST), alanine aminotransferase (ALT), and alkaline phosphatase (ALP).

Serum hormone levels of triiodothyronine (T3) and thyroxine (T4) were evaluated on Days 0 and 28 using enzyme‐linked immunosorbent assay (Elabscience ELISA kits) techniques with multispecies commercial kits, following the manufacturer's instructions.

At the end of the experimental period (29 days), the rabbits were euthanized for necropsy and histopathological evaluation. Animals from all groups were necropsied in a randomized order to minimize potential group‐related bias. The animals were anesthetized with 80 mg/kg of ketamine combined with 15 mg/kg of xylazine, administered intramuscularly. Once deep anesthesia was achieved, an additional dose equivalent to twice the anesthetic dose was administered, followed by exsanguination via cardiac puncture. Following euthanasia, the liver, kidneys, and spleen were weighed, and the relative weight (RW) was calculated (organ weight/body weight). Representative tissue samples from the thyroid, liver, kidneys, heart, lungs, and spleen were collected and fixed in formalin. The tissues were dehydrated, cleared, and embedded in paraffin. Subsequently, 5 μm histological sections were prepared using a microtome, stained with hematoxylin and eosin (H&E), and analyzed under a microscope for histopathological evaluation. Lesion severity was assessed using a semi‐quantitative grading scale (0–4), in accordance with established toxicopathology methodologies, where 0 denotes no observable change and scores from 1 to 4 represent progressively increasing severity (Mann et al. 2012).

### 2.6 Statistical Analyses

The data were analyzed using GraphPad Prism 10.2.3 software (GraphPad Software Inc., San Diego, CA, USA). Homoscedasticity was assessed using the F‐test or Bartlett's test, while normality was evaluated with the Brown–Forsythe test. Parametric data were analyzed using one‐way ANOVA, followed by Dunnett's post hoc multiple comparison test. For non‐parametric data, the Kruskal–Wallis test was used, followed by Dunn's test. Results were expressed as mean ± standard error of the mean, with differences considered statistically significant at *p* < 0.05.

## 3 Results

### 3.1 Phytochemical Evaluation of L‐Mimosine

The analysis of the sample pools of L‐mimosine extracted from *L. leucocephala* seeds showed a peak at 6.756 and a purity level of 96%.

### 3.2 Clinical, Hematological, Biochemical, and Hormonal Evaluation

None of the rabbits exhibited signs of toxicity, such as alopecia, depression, or any clinical indications of morbidity. No significant differences in body weight or weight gain were observed among the four groups. All animals consistently consumed 120 g of feed per day throughout the entire experimental period. No feed remnants were observed in the feeder during this time (Table 1; *p* > 0.05).

**TABLE 1:** Initial, final, and total body weight gain, as well as relative organ weights, of male rabbits treated or untreated with different doses of L‐mimosine administered orally for 28 consecutive days (*N* = 5; mean ± SEM).

Parameters		Control	L‐mimosine (mg/kg body weight)		
			25	40	60
Body weight					
Initial	kg	2.68 ± 0.06	2.65 ± 0.14	2.59 ± 0.15	2.56 ± 0.16
Final	kg	3.11 ± 0.08	3.24 ± 0.10	3.09 ± 0.12	2.95 ± 0.18
Gain	kg	0.43 ± 0.01	0.57 ± 0.04	0.48 ± 0.04	0.40 ± 0.03
Organ weights					
Liver	g	73.40 ± 1.26	87.34 ± 5.00	85.95 ± 2.21	75.88 ± 3.20
	%	2.38 ± 0.08	2.72 ± 0.10	2.83 ± 0.12	2.58 ± 0.06
Right Kidney	g	6.90 ± 0.35	7.6 ± 0.47	7.36 ± 0.51	7.81 ± 0.51
	%	0.22 ± 0.01	0.23 ± 0.15	0.24 ± 0.02	0.26 ± 0.02
Left Kidney	g	7.12 ± 0.29	7.50 ± 0.52	7.60 ± 0.81	7.76 ± 0.46
	%	0.22 ± 0.01	0.23 ± 0.01	0.25 ± 0.03	0.26 ± 0.02
Spleen	g	1.01 ± 0.14	1.28 ± 0.11	1.26 ± 0.18	1.41 ± 0.21
	%	0.03 ± 0.00	0.04 ± 0.00	0.04 ± 0.00	0.04 ± 0.00

The hematological and biochemical evaluation revealed variations in the assessed parameters throughout the study period. However, pre‐ and post‐treatment values remained within the expected physiological ranges and showed no relevant deviations from individual baseline values. Moreover, these fluctuations were not statistically significant between the experimental and control groups (Tables 2 and 3; *p* > 0.05). Likewise, no significant differences were observed in serum T3 and T4 levels between the groups (Table 3; *p* > 0.05).

**TABLE 2:** Hemogram and leukogram of male rabbits treated or untreated with different doses of L‐mimosine administered orally for 28 consecutive days (*N* = 5; ±SEM).

Parameters	Day	Control	L‐mimosine (mg/kg body weight)		
			25	40	60
Erythrocytes (×10^6^/m^3^)	0	4.992 ± 0.083	4.886 ± 0.25	5.21 ± 0.24	4.38 ± 0.42
	7	5.75 ± 0.22	5.26 ± 0.32	5.75 ± 0.22	5.88 ± 0.33
	14	8.83 ± 0.43	5.24 ± 0.42	5.88 ± 0.54	6.66 ± 0.46
	21	4.29 ± 0.60	4.85 ± 0.32	4.73 ± 0.27	5.30 ± 0.56
	28	5.13 ± 0.26	5.17 ± 0.35	4.76 ± 0.27	5.2 ± 0.10
Hemoglobin (g/dL)	0	11.29 ± 0.20	11.34 ± 0.70	11.13 ± 0.22	10.89 ± 0.64
	7	9.54 ± 0.29	9.74 ± 0.46	9.33 ± 0.20	9.60 ± 0.26
	14	11.05 ± 0.14	10.72 ± 0.52	11.06 ± 0.52	11.10 ± 0.15
	21	11.17 ± 0.31	11.06 ± 0.21	11.45 ± 0.60	10.63 ± 0.91
	28	11.97 ± 0.12	10.98 ± 0.48	11.20 ± 0.36	11.89 ± 0.15
Hematocrit (%)	0	30.4 ± 1.80	25.6 ± 0.6	29.6 ± 2.24	26 ± 1.87
	7	30.6 ± 1.07	30 ± 1.89	28.6 ± 1.28	31 ± 0.31
	14	32.6 ± 1.28	29.4 ± 1.16	31.6 ± 1.43	31.8 ± 0.91
	21	27 ± 1.51	28.6 ± 1.74	30.2 ± 2.17	28.8 ± 1.46
	28	31.6 ± 1.02	29.6 ± 0.81	32.8 ± 4.16	34.2 ± 0.91
MCV (fL)	0	56.16 ± 4.06	52.85 ± 2.49	56.59 ± 2.66	60.49 ± 3.78
	7	53.25 ± 2.50	57.21 ± 2.44	50.14 ± 3.56	53.31 ± 2.84
	14	57.18 ± 2.30	56.92 ± 3.03	54.84 ± 3.49	48.52 ± 3.36
	21	64.64 ± 9.83	59.27 ± 2.48	64.07 ± 3.74	56.57 ± 6.18
	28	63.54 ± 4.22	58.09 ± 3.46	69.40 ± 3.32	65.97 ± 2.86
MCH (pg)	0	23.13 ± 0.78	23.46 ± 1.87	21.49 ± 0.88	25.49 ± 1.84
	7	16.68 ± 0.50	18.20 ± 0.50	16.36 ± 0.94	16.48 ± 0.87
	14	19.61 ± 1.23	20.68 ± 0.91	19.22 ± 1.31	16.99 ± 1.25
	21	29.68 ± 5.16	23.15 ± 1.28	23.47 ± 2.05	20.90 ± 2.91
	28	23.69 ± 1.59	21.43 ± 0.92	23.74 ± 1.01	22.93 ± 0.72
MCHC (%)	0	38.35 ± 1.73	44.16 ± 1.90	38.36 ± 2.53	42.37 ± 2.60
	7	31.71 ± 1.72	33.20 ± 1.74	32.77 ± 0.90	30.96 ± 0.79
	14	34.18 ± 1.11	36.41 ± 0.52	35.00 ± 0.54	34.99 ± 0.97
	21	43.41 ± 2.37	39.11 ± 1.72	37.61 ± 1.30	36.63 ± 1.47
	28	37.33 ± 0.86	37.00 ± 1.22	35.08 ± 1.15	34.84 ± 0.57
Leukocytes (10^3^/mm^3^)	0	5000 ± 1319.84	5130 ± 148.82	5940 ± 615.10	5240 ± 705.93
	7	6932 ± 546.82	5320 ± 280.44	5730 ± 360.06	6090 ± 643.31
	14	6767 ± 705.86	5190 ± 345.47	5330 ± 481.30	4760 ± 461.08
	21	5530 ± 560.93	4500 ± 351.06	4580 ± 343.72	4560 ± 467.54
	28	4710 ± 417.55	3430 ± 321.55	3630 ± 661.74	3400 ± 384.38
Monocytes (×10^3^/mm^3^)	0	3.37 ± 0.21	5.08 ± 1.35	4.99 ± 0.69	4.55 ± 0.69
	7	5.99 ± 1.07	3.88 ± 1.21	5.26 ± 1.25	5.25 ± 0.65
	14	3.78 ± 1.51	4.01 ± 1.60	2.56 ± 0.67	3.94 ± 0.67
	21	2.40 ± 0.33	4.91 ± 1.26	3.79 ± 034	4.26 ± 0.47
	28	2.89 ± 0.68	2.44 ± 0.78	2.18 ± 0.92	2.50 ± 0.30
Limphocytes (×10^3^/mm^3^)	0	79.86 ± 12.01	66.50 ± 5.42	58.00 ± 5.95	63.81 ± 5.86
	7	88.07 ± 8.56	67.88 ± 6.12	56.78 ± 5.50	75.35 ± 7.53
	14	87.52 ± 9.85	69.20 ± 5.06	56.49 ± 7.16	66.69 ± 6.32
	21	66.33 ± 10.26	58.25 ± 4.97	51.29 ± 7.93	57.03 ± 9.86
	28	62.57 ± 8.80	47.16 ± 5.34	42.28 ± 10.89	45.47 ± 8.85
Eosinophils (×10^3^/mm^3^)	0	0 ± 0	1.37 ± 0.85	2.01 ± 0.32	1.35 ± 0.44
	7	2.08 ± 0.65	1.65 ± 0.75	2.09 ± 0.53	1.52 ± 0.47
	14	2.22 ± 0.85	2.18 ± 0.66	2.82 ± 0.68	0.35 ± 0.35
	21	1.82 ± 0.46	1.77 ± 0.59	1.14 ± 0.48	0.41 ± 0.26
	28	0.17 ± 0.17	0.16 ± 0.16	0.51 ± 0.21	0.82 ± 0.22
Neutrophils (×10^3^/mm^3^)	0	36.42 ± 1.75	29.64 ± 4.77	53.78 ± 11.34	35.07 ± 7.84
	7	38.11 ± 4.50	32.98 ± 3.39	50.45 ± 7.44	39.66 ± 6.88
	14	37.51 ± 5.65	28.39 ± 4.48	49.26 ± 10.29	21.93 ± 5.88
	21	38.02 ± 6.28	25.06 ± 2.95	35.36 ± 7.76	29.30 ± 3.10
	28	28.68 ± 4.17	18.83 ± 1.91	27.61 ± 3.87	19.19 ± 2.31
Basophils (×10^3^/mm^3^)	0, 7, 14, 21, 28	0.0 ± 0.0	0.0 ± 0.0	0.0 ± 0.0	0.0 ± 0.0

Abbreviations: MCH: mean corpuscular hemoglobin; MCHC: mean corpuscular hemoglobin concentration; MCV: mean corpuscular volume.

**TABLE 3:** Biochemical evaluation and serum Triiodothyronine (T3) and thyroxine (T4) levels in male rabbits treated or untreated with different doses of L‐mimosine administered orally for 28 consecutive days (*N* = 5; ± SEM).

Parameters	Day	Control	L‐mimosine (mg/kg body weight)		
			25	40	60
AST (U/L)	0	30.5 ± 4.27	41.09 ± 5.10	44.04 ± 19.34	37.62 ± 2.86
	14	21.62 ± 1.55	26.66 ± 3.25	30.74 ± 4.07	26.46 ± 2.59
	28	34.88 ± 3.81	49.66 ± 7.68	79.84 ± 23.27	35.78 ± 3.32
ALT (U/L)	0	34.34 ± 3.35	32.34 ± 4.87	43.48 ± 7.65	32.78 ± 4.01
	14	21.18 ± 2.78	21.88 ± 4.00	32.08 ± 3.73	18.94 ± 2.10
	28	24.38 ± 5.03	33.4 ± 4.30	52.74 ± 11.15	21.98 ± 2.02
GGT (U/L)	0	6.42 ± 0.39	8.06 ± 2.91	12.74 ± 2.37	10.32 ± 2.10
	14	9.26 ± 1.12	9.12 ± 1.19	6.06 ± 0.38	7.08 ± 0.94
	28	18.43 ± 3.39	15.27 ± 2.14	11.8 ± 1.56	12.68 ± 0.24
ALP (U/L)	0	233.2 ± 12.62	179.6 ± 25.04	215.38 ± 21.10	213.36 ± 23.72
	14	142.14 ± 7.57	129.58 ± 13.80	130.44 ± 15.85	139.28 ± 14.77
	28	143.62 ± 7.24	120.48 ± 13.59	129.88 ± 13.13	110.06 ± 11.18
Total proteins (g/dL)	0	4.94 ± 0.9	5.42 ± 0.18	6.02 ± 0.29	5.64 ± 0.13
	14	5.88 ± 0.35	5.68 ± 0.16	5.68 ± 0.21	5.98 ± 0.45
	28	5.58 ± 0.07	5.58 ± 0.27	5.76 ± 0.13	6.02 ± 0.30
Albumin (g/dL)	0	3.54 ± 0.08	3.54 ± 0.04	3.52 ± 0.08	3.66 ± 0.09
	14	3.02 ± 0.15	3.24 ± 0.06	3.26 ± 0.06	3.46 ± 0.08
	28	3.52 ± 0.08	3.4 ± 0.10	3.3 ± 0.10	3.36 ± 0.02
Glucose (mg/dL)	0	134.74 ± 4.02	150.86 ± 2.49	148.24 ± 9.29	176.32 ± 7.19
	14	117.6 ± 13.13	104.76 ± 6.90	121.36 ± 5.25	134.38 ± 8.70
	28	100.64 ± 7.01	102.6 ± 3.39	115.88 ± 3.76	109.1 ± 5.89
Cholesterol (mg/dL)	0	53.85 ± 2.85	67.58 ± 8.54	66.28 ± 1.02	68.02 ± 4.84
	14	41.92 ± 4.65	54.36 ± 9.01	49.56 ± 6.99	48.24 ± 3.78
	28	38.43 ± 3.22	52.04 ± 9.28	39.78 ± 4.85	47.08 ± 5.83
Urea (mg/dL)	0	46.5 ± 0.57	42.92 ± 2.02	41.42 ± 2.86	49.24 ± 5.27
	14	26.28 ± 2.18	29.9 ± 0.71	30.02 ± 1.02	33.48 ± 2.25
	28	32.44 ± 1.70	35.92 ± 2.09	37.86 ± 3.21	41.52 ± 2.92
Creatinine (mg/dL)	0	1.33 ± 0.06	1.42 ± 0.04	1.46 ± 0.06	1.36 ± 0.14
	14	1.46 ± 0.04	1.48 ± 0.04	1.35 ± 0.08	1.38 ± 0.08
	28	1.73 ± 0.07	1.90 ± 0.13	1.73 ± 0.09	1.77 ± 0.16
T3 (pg/mL)	0	13.78 ± 1.82	14.43 ± 1.30	12.67 ± 1.26	10.76 ± 0.96
	28	11.30 ± 0.37	15.70 ± 2.42	10.81 ± 0.92	13.2 ± 1.11
T4 (pg/mL)	0	2.67 ± 0.21	2.25 ± 0.15	2.48 ± 0.17	2.30 ± 0.63
	28	2.62 ± 0.36	2.25 ± 0.32	2.20 ± 0.17	2.95 ± 0.19

Abbreviations: ALP: alkaline phosphatase; ALT: alanine aminotransferase; AST: aspartate aminotransferase; GGT: gamma‐glutamyl transferase; T3: triiodothyronine; T4: thyroxine.

### 3.3 Necropsy and Histopathological Evaluation

The necroscopic evaluation conducted at the end of the experimental period revealed no significant macroscopic alterations. The mucosae appeared normochromic and intact. The thoracic and abdominal cavities, as well as their respective organs, exhibited normal coloration and appearance. The relative weights of the liver, kidneys, and spleen showed no significant differences between the groups (Table 1; *p* > 0.05).

**FIGURE 1:**
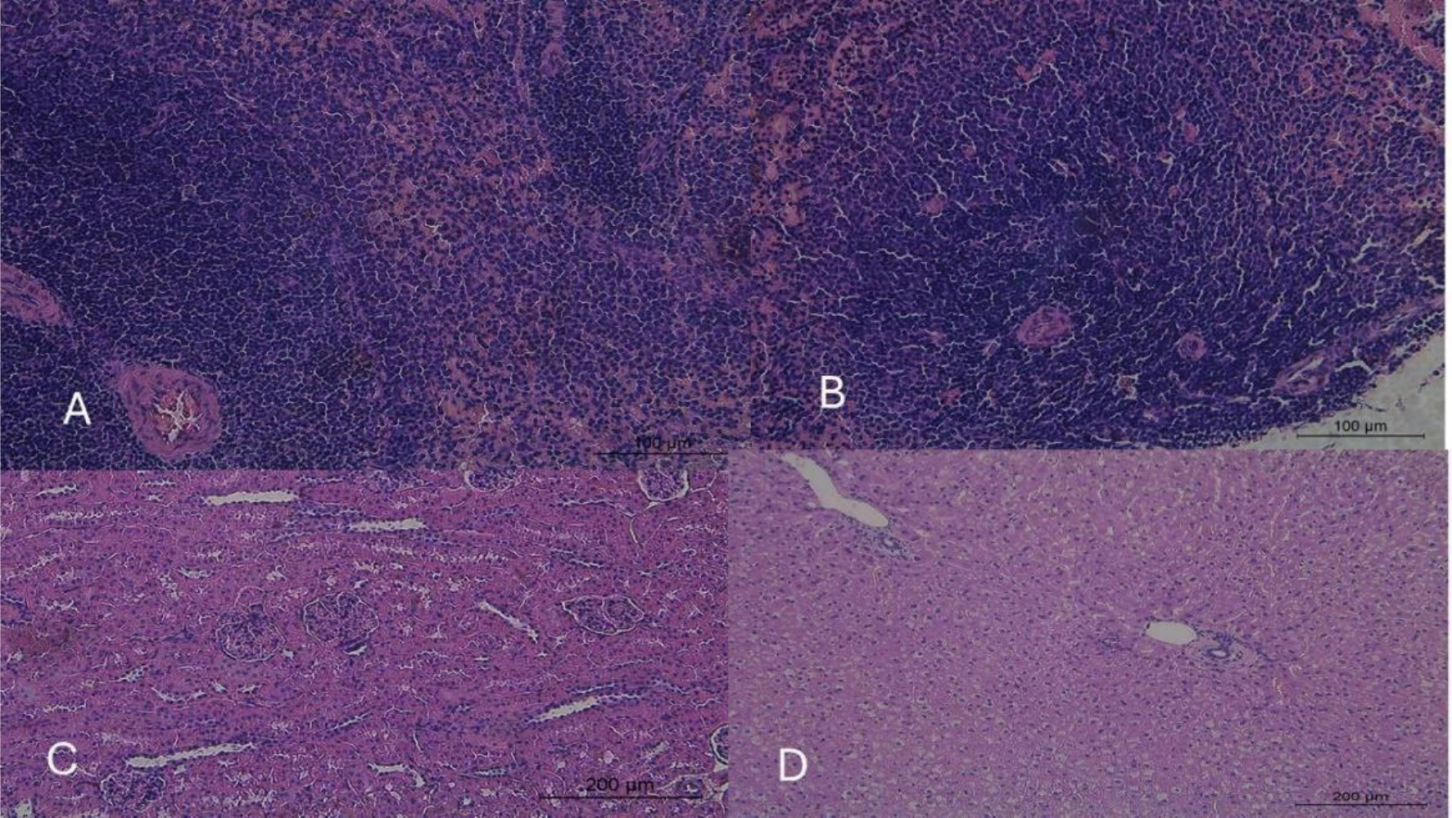
Photomicrographs of the spleen (A and B), kidney (C) and liver (D) of adult male rabbits from the control group.

**FIGURE 2:**
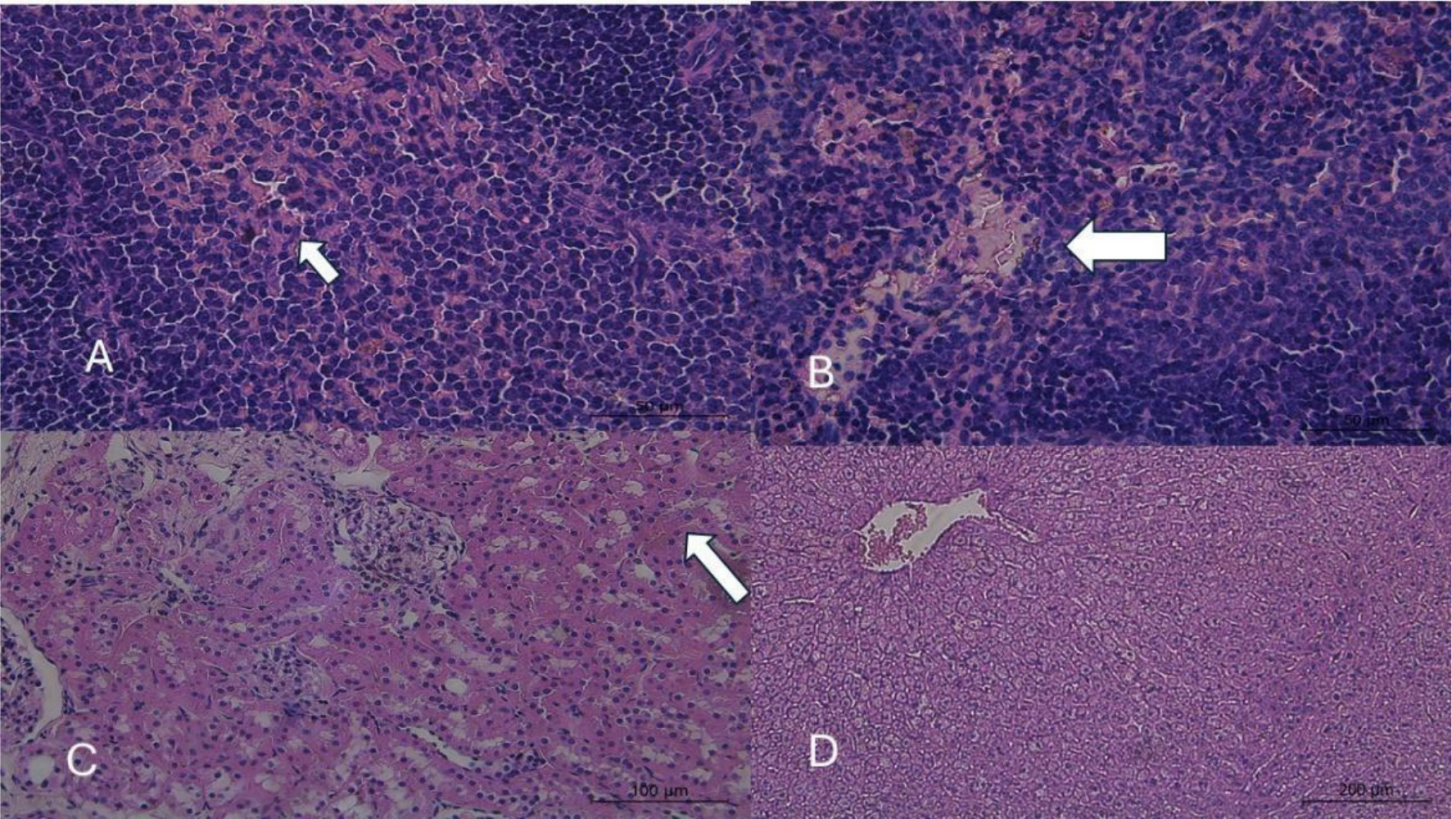
Photomicrographs of the liver, kidney, and spleen of male rabbits treated daily for 28 days with L‐mimosine incorporated into the feed at a dose of 25 mg/kg of body weight. The images highlight: (A) mild foci of congestion and hemorrhage in spleen lymphoid follicles, accompanied by hemosiderosis (indicated by the arrow); (B) foci of edema and necrosis in spleen lymphoid follicles (arrow); (C) congestion and intratubular hemorrhage foci in the kidney (arrow); and (D) mild hepatic congestion.

**FIGURE 3:**
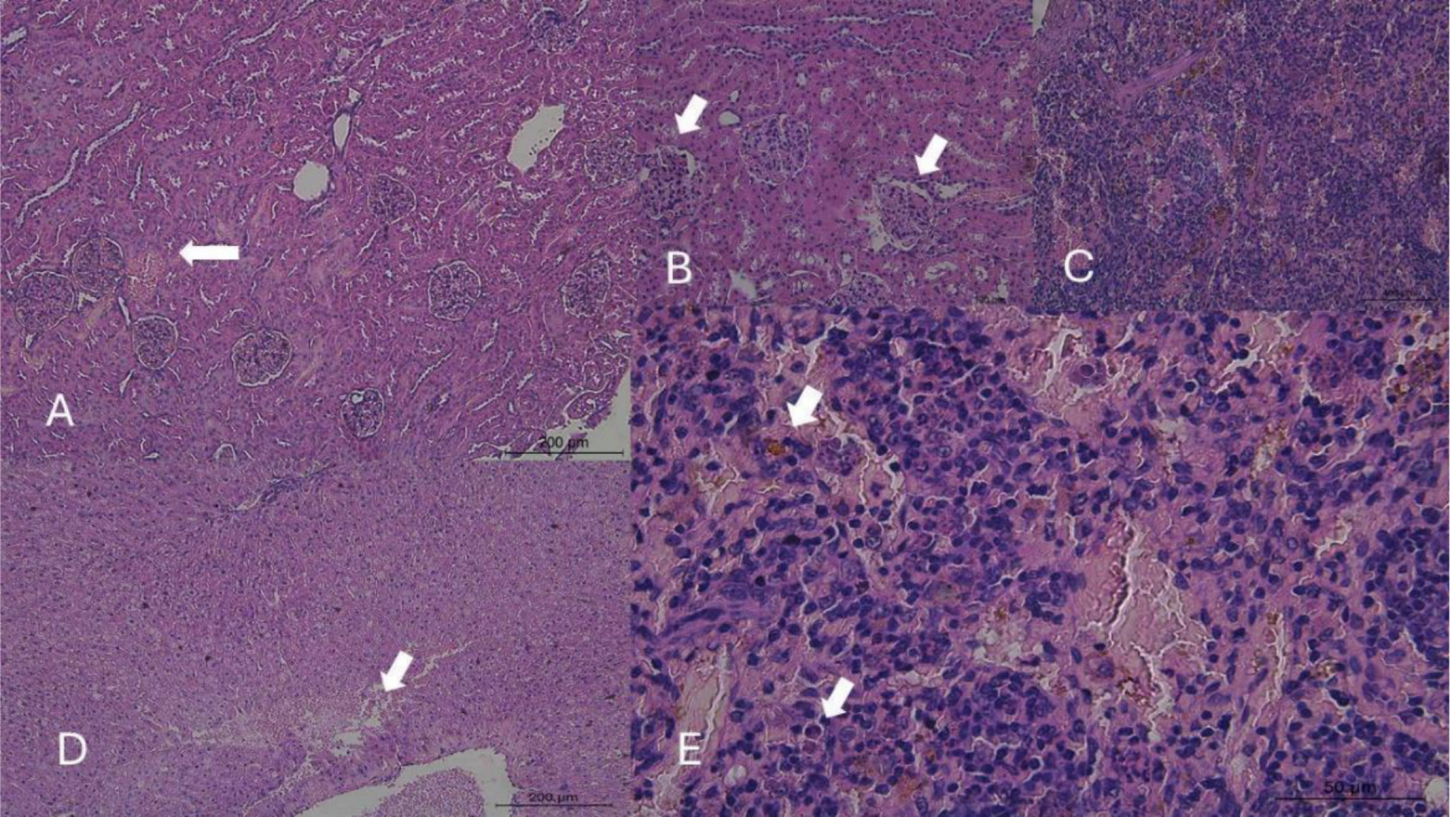
Photomicrographs of the liver, kidney, and spleen of male rabbits treated daily for 28 days with mimosine incorporated into the feed at a dose of 40 mg/kg of body weight. The images highlight the following: (A) Mild to moderate renal congestion accompanied by small foci of hemorrhage (arrow); (B) Dilation of the glomerular urinary space with the presence of red blood cells (arrows); (C) Areas of intense hemosiderosis in the spleen; (D) Foci of hemorrhage and hepatocyte vacuolization in the centrilobular zone of the liver; (E) Hemosiderosis associated with the presence of polymorphonuclear cells (arrow) in necrotic‐hemorrhagic areas of lymphoid follicles in the spleen.

**FIGURE 4:**
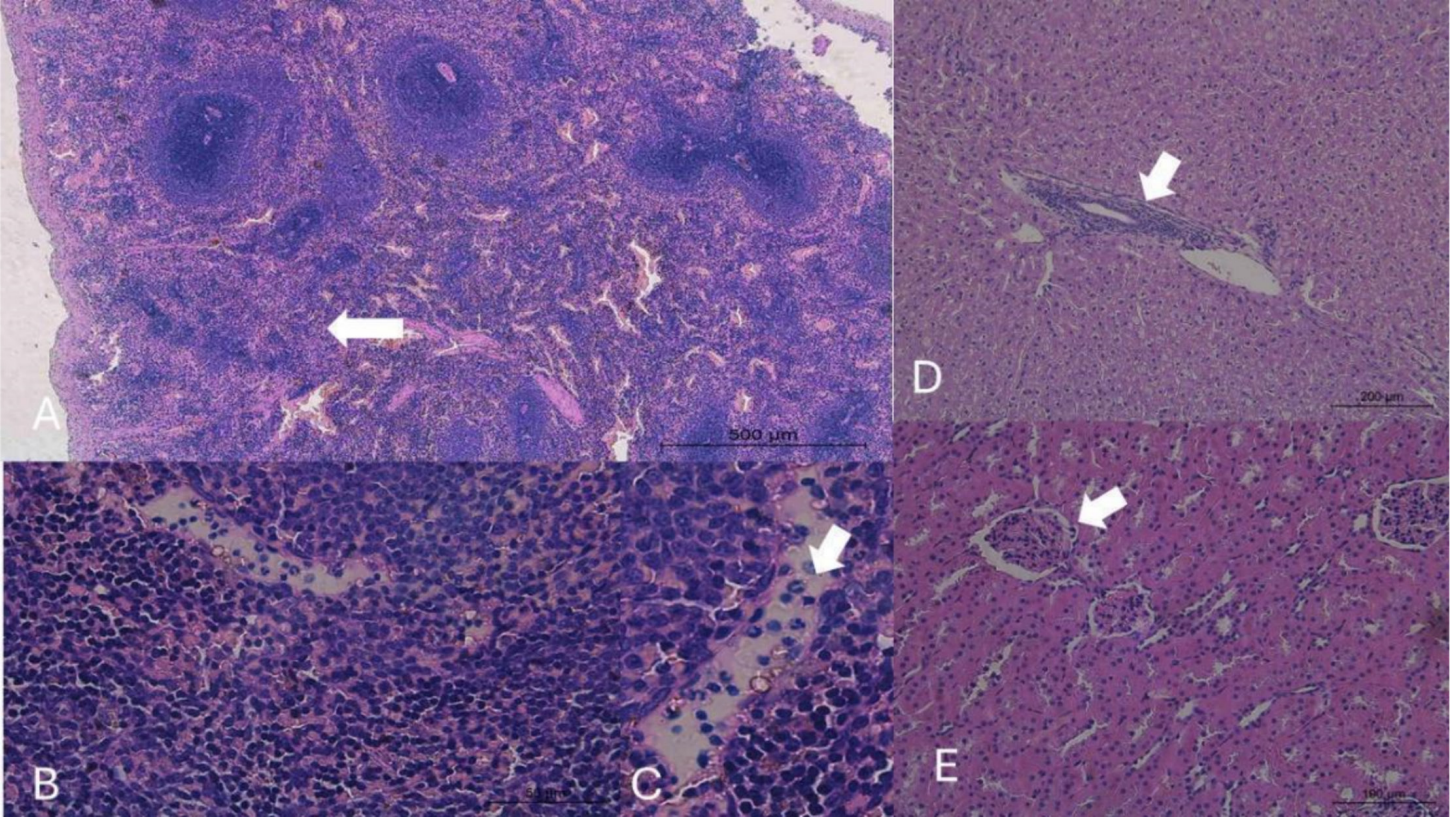
Photomicrographs of the liver, kidney, and spleen of male rabbits treated daily for 28 days with mimosine incorporated into the feed at a dose of 60 mg/kg of body weight. The images highlight the following: (A) Rarefaction and atrophy of lymphoid follicles in the spleen (arrow); (B and C) Infiltration of polymorphonuclear inflammatory cells in the lymphoid follicles of the spleen (arrow); (D) Mild congestion and hepatocyte vacuolization, associated with mononuclear infiltration around hepatic bile ducts; (E) Presence of tubular casts, marked dilation of the urinary space, congestion in glomerular tuft vessels, and loss of red blood cells.

The histopathological analysis revealed significant and dose‐dependent alterations in the liver, kidneys, thyroid, and most notably, the spleen of rabbits exposed to mimosine compared to the control group (Figures 1, 2, 3, and 4; Table 4).

**TABLE 4:** Incidence and severity grading of histopathological lesions in male rabbits orally treated with different doses of L‐mimosine for 28 consecutive days (*N* = 5 per group). Lesions graded on a 0–4 scale are indicated in parentheses.

Tissue/Parameters	Control	L‐mimosine (mg/kg body weight)		
		25	40	60
Liver:				
Congestion	5/5 (0)	4/5 (2)	5/5 (2)	4/5 (2)
Vacuolization	5/5 (0)	5/5 (0)	5/5 (1)	5/5 (2)
Hemorrhage	5/5 (0)	3/5 (0)	3/5 (1)	5/5 (2)
Mononuclear infiltration	5/5 (0)	3/5 (0)	5/5 (0)	4/5 (1)
Spleen:				
Hemosiderosis	5/5 (0)	5/5 (2)	5/5 (3)	5/5 (4)
Congestion in lymphoid follicles	5/5 (0)	4/5 (2)	5/5 (2)	5/5 (2)
Edema in lymphoid follicles	5/5 (0)	4/5 (1)	4/5 (2)	4/5 (2)
Hemorrhage in lymphoid follicles	5/5 (0)	3/5 (2)	4/5 (2)	4/5 (3)
Rarefaction of lymphoid follicles	5/5 (0)	3/5 (0)	5/5 (2)	5/5 (4)
Necrosis in lymphoid follicles	5/5 (0)	3/5 (1)	5/5 (2)	5/5 (2)
Polymorphonuclear infiltration	5/5 (0)	3/5 (0)	5/5 (2)	5/5 (3)
Kidney				
Congestion	5/5 (0)	5/5 (1)	5/5 (3)	5/5 (3)
Glomerular degeneration	5/5 (0)	5/5 (0)	4/5 (1)	5/5 (2)
Tubular degeneration	5/5 (0)	5/5 (0)	4/5 (1)	4/5 (2)
Intratubular hemorrhage	5/5 (0)	2/5 (1)	5/5 (2)	4/5 (2)
Thyroid				
Vacuolization	5/5 (0)	5/5 (1)	5/5 (2)	5/5 (3)
Colloid depletion	5/5 (0)	5/5 (1)	5/5 (3)	5/5 (4)

*Note:* Values represent semi‐quantitative lesion scores: 0 = no lesion; 1 = minimal; 2 = mild; 3 = moderate; 4 = severe. All findings were confirmed by blinded histological evaluation.

In animals that received a dose of 25 mg/kg, mild morphological changes were observed, including hepatic and renal congestion. The spleen exhibited more evident alterations, with mild hemosiderosis in the lymphoid follicles. In the group treated with 40 mg/kg, the observed changes were similar to those in the lower‐dose group but more pronounced; in the liver, centrilobular congestion, hepatocyte vacuolization, and scattered hemorrhagic foci were identified; in the spleen, necrosis and hemorrhage in the lymphoid follicles were noted, accompanied by the presence of polymorphonuclear cells. Animals treated with the highest dose (60 mg/kg) exhibited the most severe alterations in the spleen, including marked rarefaction and atrophy of the lymphoid follicles. A substantial number of polymorphonuclear cells were observed margined along capillaries surrounding necrotic foci in the lymphoid follicles. Renal alterations included tubular degeneration, the presence of proteinaceous casts, pronounced dilation of the urinary space with free erythrocytes, and congestion of glomerular vessels. In the liver, in addition to congestion and hepatocyte vacuolization, mononuclear inflammatory infiltration was observed around the bile ducts. Across all experimental groups, thyroid alterations were observed in a dose‐dependent manner, characterized by follicles exhibiting vacuolization, varying sizes, and loss of colloid staining.

## 4 Discussion

The toxicodynamics of L‐mimosine involve multiple mechanisms. As a structural analogue of tyrosine, it interferes with tyrosine‐dependent metabolic pathways, affecting enzymatic activity and cellular signaling. It also inhibits DNA synthesis by targeting ribonucleotide reductase, particularly impairing replication in highly proliferative cells. Additionally, L‐mimosine acts as a potent iron and copper chelator, disrupting their bioavailability and interfering with essential physiological processes. Furthermore, its biotransformation produces 3‐hydroxy‐4(1H)‐pyridone (3,4‐DHP), an aromatic metabolite that inhibits thyroid peroxidase, blocking tyrosine iodination and ultimately reducing thyroid hormone synthesis (Nguyen and Tawata 2016; Coy et al. 2024). These mechanisms explain the wide range of toxic effects associated with L‐mimosine.

It is well established that monogastric species, including humans and rabbits, are more sensitive to L‐mimosine toxicity than ruminants due to their lack of microbial detoxification, inefficient hindgut fermentation, and greater systemic absorption of the toxin (Dearing and Weinstein 2022). However, when considering conventional monogastric laboratory animals, a direct comparison of L‐mimosine sensitivity between rabbits and rats was not possible. For a valid comparison, key experimental parameters would have needed to be standardized, including dosage (expressed in mg/kg bw), route of administration, sex, and exposure duration (Burnett et al. 2021).

Previous studies in which growing or adult rabbits received different concentrations of *L. leucocephala* in their diet, with L‐mimosine intake ranging from 110 to 325 mg/kg bw/day over periods of 4 to 12 weeks, consistently reported reduced growth performance across all treatments (Tangendjaja et al. 1990; Gupta and Atreja 1998; Fayemi et al. 2011; Adekojo et al. 2014). Additionally, in one of these studies (Fayemi et al. 2011), rabbits that received 325 mg/kg bw/day for 10 weeks also exhibited alopecia, necrotic spots, liver congestion, edema, and the highest mortality rate.

In the present study, we selected L‐mimosine doses and the period of L‐mimosine exposure based on earlier research conducted by our group (Gotardo et al. 2011; Hueza et al. 2023), which used rats as the animal model. The chosen doses were 25, 40, and 60 mg/kg bw/day for 4 weeks. Consequently, the levels of this amino acid administered to rabbits in former studies (Tangendjaja et al. 1990; Gupta and Atreja 1998; Fayemi et al. 2011; Adekojo et al. 2014) were substantially higher than those used here. Moreover, in those studies, *L. leucocephala* was incorporated into the diet; thus, multiple active compounds may have contributed to the observed effects, rather than L‐mimosine alone, making direct comparisons with our study more complex.

In this study, although no clinical, biochemical, or necropsy findings were observed in rabbits from the different groups, histopathological analysis identified lesions in the liver, kidneys, thyroid, and most notably, the spleen of rabbits exposed to mimosine.

Although the effects of L‐mimosine have been extensively studied in animal production and rats, hepatic and renal lesions resulting from exposure to this non‐protein amino acid had not been previously reported in these species. However, the dose‐dependent lesions observed in the present study provide clear evidence of mimosine‐induced hepatic and renal toxicity, suggesting a broader organ‐specific impact of mimosine in rabbits. The harmful effects of L‐mimosine in both organs here verified agree with the report of Fayemi et al. (2011) and Adekojo et al. (2014), which observed abnormalities in the liver of rabbits fed *L. leucocephala* leaves; however, it should be considered that the calculated doses of L‐mimosine contained in the plant were at least double the dose of the amino acid employed in the present study.

The histopathological analysis in this study revealed dose‐dependent thyroid lesions in animals treated with L‐mimosine. While some authors suggest that only ruminants metabolize L‐mimosine into 3,4‐DHP, the goitrogenic metabolite, due to the presence of specialized gut microbiota (Hammond 1995; Derakhshani et al. 2016), our findings in rabbits reinforce and refine our previous study (Gotardo et al. 2011), in which we documented thyroid lesions in rats exposed to L‐mimosine. These results further support its goitrogenic potential in monogastric species.

In a previous study conducted by our group to evaluate the effects of L‐mimosine on immune responses in rats (Hueza et al. 2023) using the same doses employed in the present study, no alterations were observed in the spleen. However, a significant reduction in antibody production was detected. In contrast, a key finding in the current study was the presence of splenic degeneration, characterized by a marked reduction in lymphoid follicles. Similar alterations were recently reported by our group (de Almeida et al. 2025) in pregnant female rats treated with 240 mg/kg bw of L‐mimosine. These findings suggest that rabbits may be more sensitive to the splenic harmful effects of L‐mimosine than rats, as a substantially higher dose was required to induce morphological alterations in this organ in rodents compared to the doses used in this study.

The spleen plays a crucial role in immune function, hematopoiesis, and red blood cell clearance (Lewis et al. 2019). It is responsible for removing aged or damaged blood cells, particularly senescent erythrocytes, with splenic macrophages degrading hemoglobin and releasing iron for reutilization. The liberated iron can be stored intracellularly as ferritin or exported into the bloodstream via the iron transporter ferroportin (Kapila et al. 2023). This process suggests that the spleen contains a high concentration of free iron ions. Given that L‐mimosine is a well‐known iron chelator and its complexation with iron can induce oxidative stress, contributing to cytotoxic effects, the spleen emerges as a relevant candidate for L‐mimosine toxicity.

It is important to note that the present study was conducted exclusively in male animals. This decision aimed to minimize biological variability and enable a clearer interpretation of dose–response relationships during this initial phase. However, sex is a well‐established biological variable that can significantly influence toxicokinetics and toxicodynamics, including differences in the absorption, distribution, metabolism, and excretion of xenobiotics, as well as in target organ susceptibility (Soldin and Mattison 2009). Such sex‐based differences can lead to distinct toxicological outcomes even under identical exposure conditions. For instance, Masubuchi et al. (2011) reported greater susceptibility to acetaminophen‐induced hepatotoxicity in male CD‐1 mice compared to females. Therefore, future studies are warranted to determine whether female rabbits exhibit differential responses to L‐mimosine, which could further clarify the species‐ and sex‐specific sensitivity to this compound.

The present study provides new insights into the toxicological effects of L‐mimosine in rabbits, highlighting its organ‐specific impact. Although no clinical, biochemical, or macroscopic alterations were observed, histopathological findings revealed dose‐dependent lesions in the liver, kidneys, thyroid, and spleen. Furthermore, the dose‐dependent thyroid lesions confirm its goitrogenic potential in monogastric animals, reinforcing previous findings in rats. The observed splenic degeneration, characterized by a marked reduction in lymphoid follicles, suggests a possible immunosuppressive effect of L‐mimosine in rabbits, which appears to be more pronounced than in rats. Collectively, these findings suggest that rabbits may be particularly vulnerable to its toxic effects.

Future studies will include histopathological evaluation of the brain, peripheral nerves, eyes, reproductive organs, gastrointestinal tract, and bone marrow, as well as determination of L‐mimosine blood concentrations, to provide a more comprehensive assessment of its toxicity in rabbits. In addition, studies should evaluate the reproductive toxicity and immunotoxic effects of L‐mimosine, further exploring the susceptibility of rabbits as a model for L‐mimosine intoxication.

## Author Contributions

S.L.G. and A.T.G. conceived and designed the experiments; S.M.F. and C.S.M. performed the experiments; S.M.F., H.Z., C.S.M., P.C.M., and A.T.G. analyzed the samples; A.T.G. and A.F.C.A. analyzed the data; S.M.F., H.Z., S.L.G., and A.T.G. wrote the paper.

## Ethics Statement

The procedures were approved by the School of Veterinary Medicine and Animal Sciences, USP Animal Ethics Committee (protocol number CEUA 8425111022).

## Conflicts of Interest

The authors declare no conflicts of interest.

## Data Availability

The data that support the findings of this study are available from the corresponding author upon reasonable request.
